# Effect of cryosurgery on liver blood flow.

**DOI:** 10.1038/bjc.1993.278

**Published:** 1993-07

**Authors:** N. J. Brown, P. Bayjoo, M. W. Reed

**Affiliations:** Department of Surgical and Anaesthetic Sciences, University of Sheffield, UK.

## Abstract

The effect of cryosurgery on normal liver and liver tumour was investigated using 60 adult male rats. Animals were divided into four groups Group A implanted tumour/cryosurgery (n = 19), Group B normal liver/cryosurgery (n = 17), Group C normal liver/sham cryosurgery (n = 10) and Group D implanted tumour/sham cryosurgery (n = 14). At laparotomy animals were injected into the left lateral lobe of the liver with 10(5) HSN fibrosarcoma cells or vehicle. Two weeks after implantation red cell flux was recorded in all animals, the appropriate groups treated with cryosurgery and after thawing red cell flux was monitored over the tumour and at the edge of the cryolesion and over the corresponding normal area in controls. In certain animals red cell flux was measured at hourly intervals for 8 h, and in further groups at 24 h and at 2 weeks after cryosurgery. Results demonstrated that cryosurgery significantly reduced (P < 0.01) red cell flux in both normal and tumour liver, immediately after treatment. Red cell flux remained significantly reduced (P < 0.005) at 8 h after treatment but by 24 h had returned to preoperative levels which was maintained at 2 weeks. The results suggest that microcirculatory shutdown may be a contributing factor to the tumour necrosis occurring after cryosurgery.


 © Macmillan Press Ltd., 1993

SHORT COMMUNICATION

### Effect of cryosurgery on liver blood flow

N.J. Brown, P. Bayjoo & M.W.R. Reed

*Department of Surgical and Anaesthetic Sciences, The University of Sheffield, Floor K, Royal Hallamshire Hospital, Glossop Road, Sheffield, S10 2JF, UK.*

**Summary** The effect of cryosurgery on normal liver and liver tumour was investigated using 60 adult male rats. Animals were divided into four groups Group A implanted tumour/cryosurgery ($n = 19$), Group B normal liver/cryosurgery ($n = 17$), Group C normal liver/sham cryosurgery ($n = 10$) and Group D implanted tumour/sham cryosurgery ($n = 14$). At laparotomy animals were injected into the left lateral lobe of the liver with $10^5$ HSN fibrosarcoma cells or vehicle. Two weeks after implantation red cell flux was recorded in all animals, the appropriate groups treated with cryosurgery and after thawing red cell flux was monitored over the tumour and at the edge of the cryolesion and over the corresponding normal area in controls. In certain animals red cell flux was measured at hourly intervals for 8 h, and in further groups at 24 h and at 2 weeks after cryosurgery. Results demonstrated that cryosurgery significantly reduced ($P < 0.01$) red cell flux in both normal and tumour liver, immediately after treatment. Red cell flux remained significantly reduced ($P < 0.005$) at 8 h after treatment but by 24 h had returned to preoperative levels which was maintained at 2 weeks. The results suggest that microcirculatory shutdown may be a contributing factor to the tumour necrosis occurring after cryosurgery.

Cryosurgery is the use of freezing to destroy living tissues and has been used in the treatment of liver metastases from colo-rectal carcinoma (Charnley *et al.*, 1989). It is thought that cryosurgery causes tumour necrosis due to the formation of ice crystals in the cells (Whittaker, 1984). Previous studies in our laboratory have demonstrated that in liver with implanted HSN fibrosarcoma, treatment with cryosurgery results in tumour destruction with no evidence of regrowth at 6 weeks (Bayjoo, 1992). If the tumour was excised immediately after treatment, tumour cells were viable and could be grown in culture *in vitro* (Bayjoo, 1992) implying that an additional secondary host factor may contribute to the tumour destruction. The studies also identified alterations in the immunological status up to 11 days after treatment. Natural killer (NK) cells from peripheral blood were found to have increased cytotoxic potential *in vitro* whereas NK cells of splenic origin had decreased cytotoxic potential *in vitro* (Bayjoo *et al.*, 1991). However little attention has been given to the local effects of cryosurgery on the tumour blood supply. It has been shown that large blood vessels may act as a heat sink to freezing (Gage *et al.*, 1985; McIntosh *et al.*, 1985) while smaller vessels such as arterioles and venules are less resistant with evidence of damage and gap formation in the endothelium cryosurgery resulting in extravasation of intraluminal contents (Whittaker, 1984).

Based on these observations, it was hypothesised that cryosurgery may result in a reduction in blood flow in normal or tumour-bearing liver by disruption of the microcirculation resulting in hypoxia. The aim of the study therefore was to monitor blood flow in normal liver and in an implanted sarcoma before and up to 2 weeks after cryosurgery using Laser Doppler Flowmetry (LDF).

### Material and methods

### *Animals*

Experiments were carried out on a total of 60 adult male albino rats, obtained from Sheffield Field Laboratories weighing between 250-300 g. All experimentation was approved by the Home Office.

Correspondence: N.J. Brown.
Received 25 September 1992; and in revised form 6 January 1993.

### *Experimental groups*

Animals were divided into four treatment groups: Group A - implanted tumour/cryosurgery ($n = 19$); Group B - normal liver/cryosurgery ($n = 17$); Group C - normal liver/sham cryosurgery ($n = 10$); Group D - implanted tumour/sham cryosurgery ($n = 14$).

### *Tumour implantation*

All animals underwent an initial laparotomy under ether anaesthesia. Groups A and D animals each received an inoculation of $10^5$ HSN fibrosarcoma cells (Currie & Gage, 1973) suspended in phosphate buffered saline (PBS) into the left lateral lobe of the liver. Groups B and C each received a control inoculation of 50 µl of PBS into the same lobe. The abdomen was closed and the animals allowed to recover.

### *Experimental protocol*

Fourteen days after tumour implantation, a second laparotomy under hypnorm/diazepam (1:1 v/v subcutaneous s.c.) anaesthesia was performed in all groups (A-D) of animals. In each animal, red cell flux (RCF) was measured with the Periflux PF 2B laser doppler flowmeter using 4 KHz bandwidth and 1.5 ms time constant. The probe was positioned on the surface of the tissue using a micromanipulator.

Group A animals underwent cryosurgery of the implanted HSN fibrosarcoma tumour. Group B animals underwent cryosurgery of a corresponding area of normal liver. Group C animals had sham cryosurgery of normal liver. This consisted of placing the probe over the liver surface without switching the machine on for a similar period of time to the treatment groups A and B. Similarly Group D animals underwent sham cryosurgery of liver tumours. Red cell flux readings were taken at the centre and the edge of the frozen area in Groups A and B and at equivalent sites in Groups C and D. Red cell flux was measured in all animals immediately before and 5 min after cryosurgery when the frozen area had thawed, or after sham treatment. Measurements of red cell flux were taken again 2 weeks after treatment prior to killing the animals by cervical dislocation.

Further experiments were performed to study in more detail the effects of cryosurgery in the first 24 h after cryosurgery. In some animals from Groups A and B red cell flux was recorded hourly for 8 h, whilst further animals from all groups were studied at 24 h.

Cryosurgery was performed using the CS-76 Frigitronics cryounit. The cryo-probe was placed in contact with the liver tumour or normal tissue and freezing continued until the freezing edge advanced well beyond the margin of the study area. The tissue was allowed to thaw and then frozen again – double freeze thaw cycle. Throughout the studies a double freeze thaw cycle was used since this has been demonstrated to be more damaging than a single cycle (Neel *et al.*, 1971*c*) possibly due to the increase in thermal conductivity following the initial freeze which makes subsequent cycles more effective (Whittaker, 1984). Sham cryosurgery consisted of the same protocol without freezing the tissue.

*Statistical analysis*

Wilcoxon signed rank test for non-parametric data was used to analyse paired data (within group comparison) and the Mann Whitney U test for non-parametric data to analyse unpaired data (between group comparison).

### Results

Before cryosurgery there were no significant differences in red cell flux between any of the study groups (Table I); red cell flux was greater in the tumour than normal liver but this did not reach statistical significance. However immediately after cryosurgery in Groups A and B, red cell flux in the treated areas was almost zero ($P<0.01$; Table I). The degree of reduction in red cell flux was similar in both tumour and normal liver after cryosurgery. Sham cryosurgery had no effect on red cell flux in either groups C or D (Table I).

Eight hours after treatment red cell flux in Groups A and B remained significantly reduced ($P<0.005$; Figure 1). However there was more variation in red cell flux in the tumour group than in the normal liver. Twenty-four hours after cryosurgery, red cell flux in Groups A and D had returned to pre-operative levels and were similar to red cell flux in untreated animals and remained normal at 2 weeks in all groups (Table I). There was no difference between red cell flux from the centre or the edge of the frozen area in any of the groups or the times recorded (Table I).

At 2 weeks tumours were noted to have been destroyed completed in all cryosurgery groups and replaced by a shallow scar. Similar lesions were noted in the normal livers treated by cryosurgery.

### Discussion

Red cell flux measured using the laser doppler flowmeter (Tenland, 1982) has been validated both theoretically and experimentally by comparison with more conventional methods of measuring blood flow such as hydrogen gas clearance (Gana *et al.*, 1987). The data demonstrate that cryosurgery produces a substantial reduction in blood flow immediately after freezing. This reduction is similar in both tumour and normal liver and is still present 8 h after cryosurgery. Furthermore by 24 h, the blood flow to the tumour and cryolesion had returned to pre-operative levels and was maintained at 2 weeks.

Cells suspensions of implanted tumours from rat livers removed immediately after cryosurgery were found to contain very few viable cells assessed using trypan blue exclusion. However trypan blue uptake is not an infalable indicator of cell death. It has been well described that trypan blue exclusion does not only indicate cell death, but also unhealthy cells (Jacob *et al.*, 1985). The ultimate test of viability is the ability of cells to grow in cell culture and to become established in a new host. These tumour cells were

**Figure 1** % Red cell flux (RCF; O) pre and post cryosurgery in two groups of animals with and without liver tumour. $n = 5$ at all treatment times, bars represent mean % red cell flux. All treatment groups are significantly different ($P<0.05$) from the pretreatment red cell flux in both groups A and B.

**Table I** Effect of cryosurgery on liver and tumour red cell flux

| | | *Group A* | *Group B* | *Group C* | *Group D* |
|---|---|---|---|---|---|
| Before cryo | T | 81 ± 5 (19) | 79 ± 7 (17) | 87 ± 7 (10) | 92 ± 5 (14) |
| | C | 84 ± 7 (19) | 81 ± 6 (17) | 86 ± 7 (17) | 91 ± 4 (14) |
| After cryo | T | 11 ± 5[a] (19) | 0[a] (17) | 82 ± 6 (10) | 93 ± 4 (14) |
| | C | 15 ± 9[a] (19) | 5 ± 5[a] (17) | 81 ± 6 (10) | 92 ± 5 (14) |
| 24 h | T | 82 ± 10 (8) | 0[a] (5) | 90 ± 5 (5) | 90 ± 8 (8) |
| | C | 76 ± 10 (8) | 0[a] (5) | 85 ± 7 (5) | 90 ± 4 (8) |
| 2 weeks | T | 86 ± 3 (6) | 95 ± 3 (6) | 71 ± 6 (10) | 88 ± 5 (6) |
| | C | 51 ± 19 (6) | 95 ± 6 (6) | 72 ± 8 (10) | 84 ± 8 (6) |

Results are expressed as mean ± s.e.m. % red cell flux with the number in parentheses representing the number of animals in each group. Group A – implanted liver tumour/cryosurgery ($n = 19$). Group B – normal liver/cryosurgery ($n = 17$). Group C – normal liver/sham cryosurgery (n = 10). Group D – implanted liver tumour/sham cryosurgery ($n = 14$). T = centre of tumour or corresponding region in non tumour-bearing animals. C = edge of frozen area or corresponding region in sham cryosurgery animals. mean ± s.e.m., [a]$P<0.05$ using Wilcoxon test comparing red cell flux at various times after cryosurgery to values obtained before cryosurgery.

cultured *in vitro* and were viable after 4 days of incubation. New host animals received tumour cell suspension from animals which had undergo cryosurgery, in 50% of animals a liver tumour had developed within 4 weeks of implantation. However tumours left *in situ* underwent necrosis (Bayjoo, 1992). While cryosurgery undoubtedly kills a large proportion of cells and damages the remainder, a further host factor may contribute to cell death *in vivo* after cryosurgery. The microcirculatory shutdown reported here may cause a reduction in tissue oxygenation for a sufficient length of time to result in cell death. It may be that freezing causes cytotoxic damage which is rendered irreversible by the subsequent microcirculatory shutdown.

The importance of the effect of cryosurgery in normal liver is demonstrated by the necessity of the cryolesion to include a rim of normal liver beyond the tumour without which tumour recurrence is inevitable. This may be due to the microscopic extension of the lesion but may also reflect the significance of vascular shutdown in the vessels from which the tumour derives its blood supply.

It is also important to state that although the red cell flux as measured using laser doppler flowmetry was equal in tumour and normal liver this does not necessarily imply that blood flow in these two very different tissues are similar (Smits *et al.*, 1986).

In conclusion, cryosurgery of normal liver and implanted tumour in the rat produces a sharp reduction in the microvascular blood flow in the treated area. This reduction in flow is maintained for at least 8 h but reversed by 24 h. This effect may contribute to the mechanism of action of cryosurgery in causing cell death.

We gratefully acknowledge Mrs B. Fairburn for culturing the tumour cells and Trent Regional Health Authority for funding this project.

### References

BAYJOO, P. (1992). *Cryosurgery and the immune system.* MD Thesis submitted to University of Leeds.

BAYJOO, P., REES, R.C., GOEPEL, J.R. & JACOB, G. (1991). The effect of cryosurgery of liver tumour on natural killer cell activity in the rat. *Int. J. Immunopath. Pharm.*, **4**, 187–190.

CHARNLEY, R.M., DORAN, J. & MORRIS, D.L. (1989). Cryotherapy for liver metastases: a new approach. *Br. J. Surg.*, **76**, 1040–1041.

CURRIE, G.A. & GAGE, J.O. (1973). Influence of tumour growth on the evolution of cytotoxic lymphoid cells in rats bearing a spontaneously metastasizing syngeneic fibrosarcoma. *Br. J. Cancer.*, **28**, 136–146.

GAGE, A.A., GUEST, K., MONTES, M., CARUANA, J.A. & WHALEN, D.A. (1985). Effect of varying freezing and thawing rates in experimental cryosurgery. *Cryobiol.*, **22**, 175–182.

GANA, T.J., HUHLEWYCH, R. & KOO, J. (1987). Focal gastric mucosal blood flow by laser-Doppler and hydrogen gas clearance: a comparative study. *J. Surg. Res.*, **43**, 337–343.

HOROWITZ, N.H., GOKALP, H. & RANDALL, J. (1966). *In situ* freezing of the common carotid artery and sagittal sinus of the dog. *Cryobiol.*, **2**, 223–228.

JACOB, G., KURZER, M.N. & FULLER, B.J. (1985). An assessment of human cell viability after *in vitro* freezing. *Cryobiology*, **22**, 417–426.

MCINTOSH, G.S., HOBBS, E.F. & O'REILLY, A.P. (1985). *In situ* freezing of the pancreas and portal vein in the pig. *Cryobiol.*, **22**, 183–190.

NEEL, H.B., KETCHAM, A.S. & HAMMOND, W.G. (1971*c*). Requisites for successful cryogenic surgery of cancer. *Arch. Surg.*, **102**, 45–48.

SMITS, C., ROMAN, R.J. & LOMBARD, J.H. (1986). Evaluation of laser doppler flowmetery as a measure of tissue blood flow. *J. Appl. Physiol.*, **61**, 666–672.

TENLAND, T. (1982). *On laser doppler flowmetry. Methods and microvascular applications.* Linkoping University Medical Dissertations.

WHITTAKER, D.K. (1984). Mechanisms of tissue destruction following cryosurgery. *Ann. RCS Eng.*, **66**, 313–318.


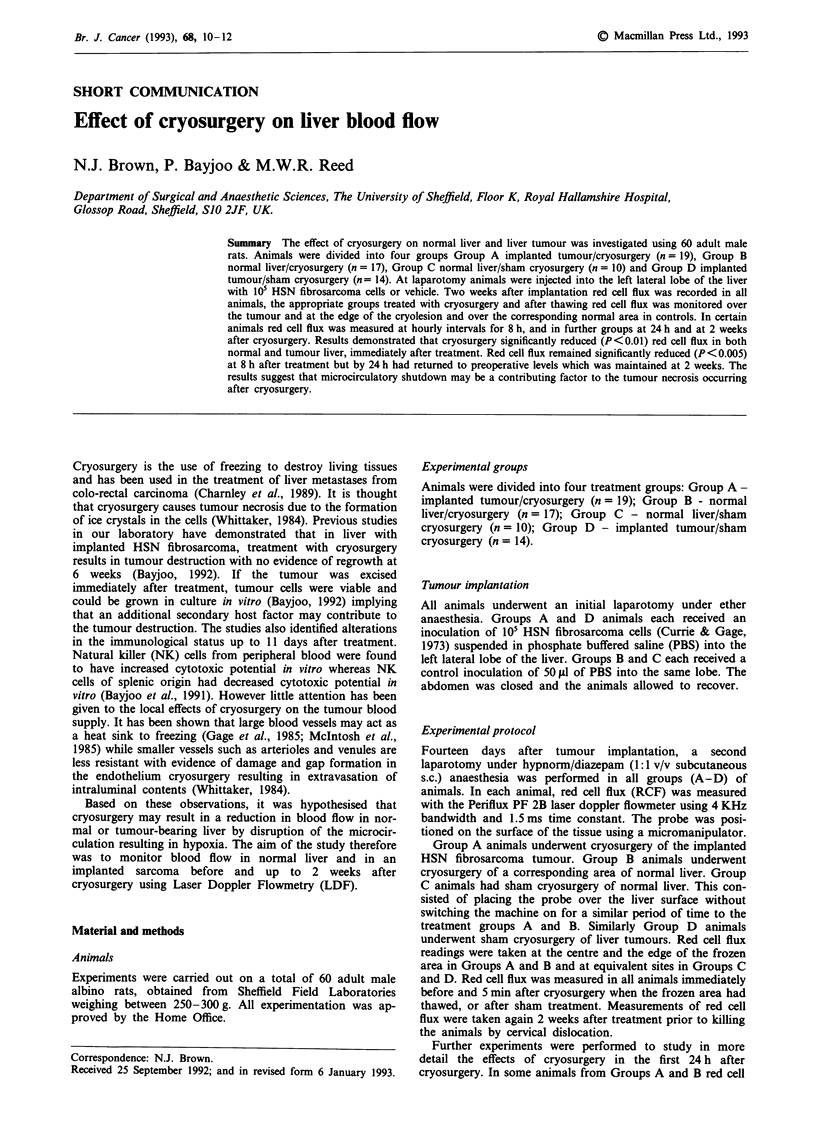

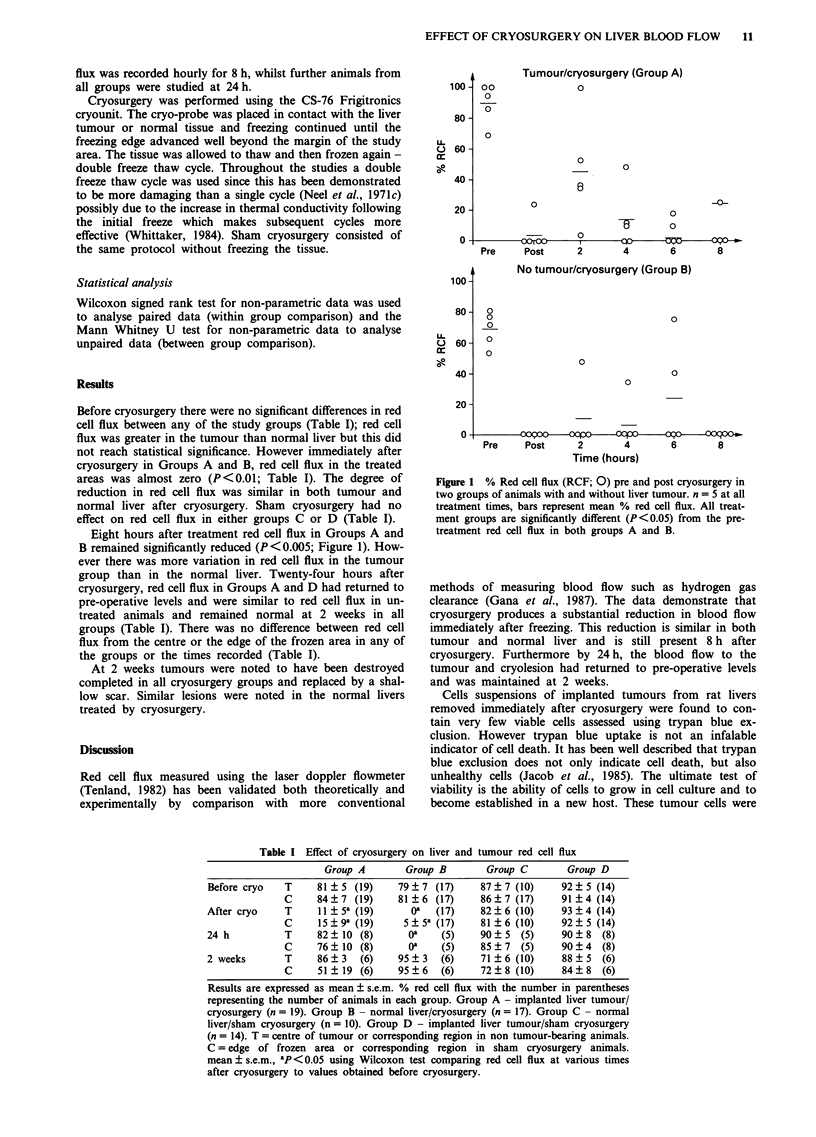

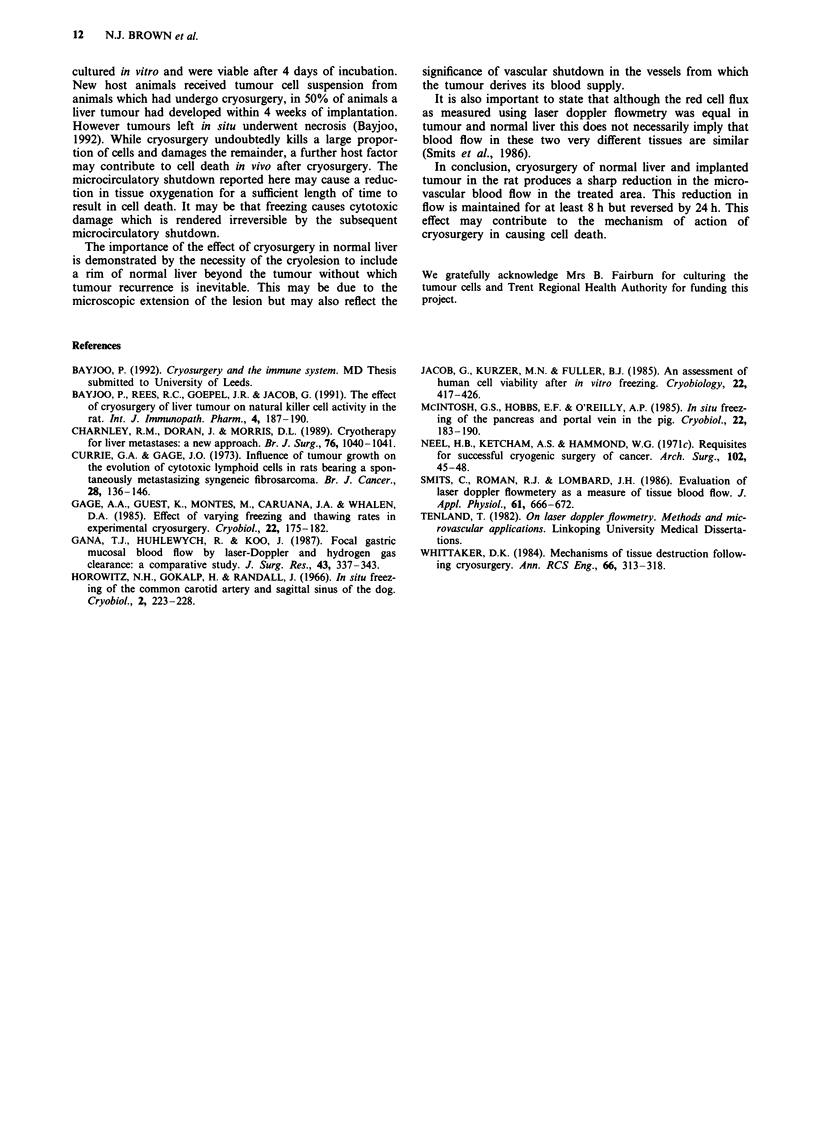


## References

[OCR_00368] Charnley R. M., Doran J., Morris D. L. (1989). Cryotherapy for liver metastases: a new approach.. Br J Surg.

[OCR_00371] Currie G. A., Gage J. O. (1973). Influence of tumour growth on the evolution of cytotoxic lymphoid cells in rats bearing a spontaneously metastasizing syngeneic fibrosarcoma.. Br J Cancer.

[OCR_00377] Gage A. A., Guest K., Montes M., Caruana J. A., Whalen D. A. (1985). Effect of varying freezing and thawing rates in experimental cryosurgery.. Cryobiology.

[OCR_00382] Gana T. J., Huhlewych R., Koo J. (1987). Focal gastric mucosal blood flow by laser-Doppler and hydrogen gas clearance: a comparative study.. J Surg Res.

[OCR_00387] Horwitz N. H., Gokalp H., Randall J. (1966). In situ freezing of the common carotid artery and sagittal sinus of the dog.. Cryobiology.

[OCR_00392] Jacob G., Kurzer M. N., Fuller B. J. (1985). An assessment of tumor cell viability after in vitro freezing.. Cryobiology.

[OCR_00397] McIntosh G. S., Hobbs K. E., O'Reilly A. P. (1985). In situ freezing of the pancreas and portal vein in the pig.. Cryobiology.

[OCR_00402] Neel H. B., Ketcham A. S., Hammond W. G. (1971). Requisites for successful cryogenic surgery of cancer.. Arch Surg.

[OCR_00407] Smits G. J., Roman R. J., Lombard J. H. (1986). Evaluation of laser-Doppler flowmetry as a measure of tissue blood flow.. J Appl Physiol (1985).

[OCR_00417] Whittaker D. K. (1984). Mechanisms of tissue destruction following cryosurgery.. Ann R Coll Surg Engl.

